# Exploring physical activity preferences and motivation in long-term cardiac prevention: An Austrian cross-sectional survey

**DOI:** 10.1371/journal.pone.0302226

**Published:** 2024-05-16

**Authors:** Hannah McGowan, Johanna Gutenberg, Veronika Leitner, Kathrin Mühlhauser, Aliz Breda, Michael Fischer, Sebastian Globits, Vincent Grote, David Kiesl, Karl Mayr, Michael Muntean, Andrea Podolsky, Josef Niebauer, Rik Crutzen, Stefan Tino Kulnik

**Affiliations:** 1 Ludwig Boltzmann Institute for Digital Health and Prevention, Salzburg, Austria; 2 Care and Public Health Research Institute, Maastricht University, Maastricht, The Netherlands; 3 Reha-Klinik Montafon, Schruns, Austria; 4 VAMED Rehabilitation Center Kitzbühel, Kitzbühel, Austria; 5 Ludwig Boltzmann Institute for Rehabilitation Research, Wien, Austria; 6 Herz-Kreislauf-Zentrum Groß Gerungs, Groß Gerungs, Austria; 7 CARDIOMED Cardiac Rehabilitation Center, Linz, Austria; 8 Department of Internal Medicine I for Hematology with Stem Cell Transplantation, Hemostaseology and Medical Oncology, Ordensklinikum Linz Elisabethinen, Linz, Austria; 9 Medical Faculty, Johannes Kepler University Linz, Linz, Austria; 10 Humanomed Center Althofen, Althofen, Austria; 11 Institute of Preventive and Applied Sports Medicine, University Hospital Krems, Karl Landsteiner University of Health Sciences, Krems, Austria; 12 University Institute of Sports Medicine, Prevention and Rehabilitation and Research Institute of Molecular Sports Medicine and Rehabilitation, Paracelsus Medical University, Salzburg, Austria; The University of Tokyo, JAPAN

## Abstract

Cardiac rehabilitation (CR) patients often do not sustain physical activity (PA) behaviour in the long run, once they progress into a self-management stage of secondary prevention. This study aimed to explore former CR patients’ PA preferences, determinants (i.e., influencing factors) and motivation for sustained PA engagement. We conducted a cross-sectional multi-centre survey using an original questionnaire based on prior qualitative interviews with cardiac patients. Five CR centres in Austria posted 500 questionnaires to former CR patients who had completed CR approximately three years prior, and 117 patients (23%) responded. Descriptive analysis was used to analyse closed-ended questions, and self-determination theory (SDT) was applied as a qualitative framework to analyse open-ended questions concerning motivation for PA engagement. Patients were generally physically active, but the majority (75.3%) did not fulfil the World Health Organisation’s recommendations for aerobic PA and muscle strengthening. Most patients preferred being physically active outdoors (70%), engaging in aerobic-related (95%), individual and non-competitive exercises, with cycling (52%), walking (32%) and hiking (25%) among the most popular activities. Main determinants of PA were health, pain and motivation for 80%, 68%, 67% of patients, respectively. A subset of patients (77%) expanded on their motivations behind PA. According to SDT, most reasons (90%) were regulated by autonomous motivation (either extrinsically autonomously-regulated or intrinsic motivation) and stemmed mostly from health-related goals (e.g., fitness, general health, weight control), future quality-of-life aspirations (e.g., self-sufficiency in old age, presence for loved ones, preserving mobility) and enjoyment of PA. Patients’ responses underscore the importance of promoting not only general PA, but also muscle strengthening training in CR interventions to maximise optimal health benefits. Our data further suggest that interventions which are aligned to patients’ health goals and foster autonomous motivation may be particularly beneficial in increasing adherence to PA in the long-term.

## Introduction

The incidence of cardiovascular disease (CVD) has nearly doubled worldwide in the past thirty years (1990–2019), and CVD remains the leading cause of death globally, including Austria [1–3]. Life-style modifications, in particular physical activity (PA) engagement, has been reported as an effective secondary prevention measure for cardiac conditions [4]. Increased PA is associated with reduced mortality risk and improved health-related quality of life [5,6]. Despite this, a survey across 27 European countries reported that two-thirds of coronary artery patients do not reach the recommended guidelines for PA [7].

Intervention strategies focused on increasing sustained PA adherence are, thus, increasingly important in the domain of secondary prevention. Although cardiac rehabilitation (CR) programs are beneficial in initiating immediate PA behaviour change, they often do not result in long-term adherence, as patients have difficulty maintaining behaviour change [8–11]. The premise that adapting PA interventions to the patient’s preferences and needs may increase adherence has been explored in the context of CR programs [12–14]. Less attention, however, has been focused on PA preferences after CR (e.g., activity types, location) when patients move from a structured and supervised CR programme to a self-management phase of care. Insight into PA preferences in this stage of prevention may be useful for transitioning PA behaviours into daily habits at the end of CR *or* for developing PA initiatives in later stages of secondary prevention.

Additionally, understanding determinants of long-term PA adherence may allow health care professionals (HCPs) to optimise PA integration into patients’ daily lives. A variety of factors are associated with PA adherence among cardiac patients, including demographics (e.g., age, gender, education), psychological factors (e.g., distress, lack of energy, well-being, self-efficacy, motivation), environment (e.g., weather, nature), physical (e.g., health), social support (e.g., family, friends, peers) and health beliefs (e.g., knowledge, awareness of threat) [15–19]. Among those factors, motivation is expressed to play a key role in PA behaviour, specifically in the context of PA maintenance [20].

In understanding motivation for PA, self-determination theory (SDT)–a well-developed psychological theory stipulating motivation exists in various forms–has extensively been applied to the domain of PA motivation [21]. Specifically, SDT focuses primarily on the type of motivation versus the degree of motivation present, and is largely driven by a distinction between self-determined or autonomous motivation and controlled motivation [22,23] The former refers to motivation that is self-endorsed, i.e., self-regulated and aligned with an individual’s personal values or interests, which can be driven by both extrinsic factors (e.g., positive health outcomes) or by intrinsic factors, an innate joy of engaging in an activity. The latter refers to motivation that is solely extrinsically regulated and lacks volition or alignment with self, where behaviour is engaged in either to fulfil others’ demands or out of avoidance of negatively experienced consequences from others (e.g., feelings of shame, guilt) [22,23]. A third type of motivation defined by SDT is amotivation, which relates to someone unwilling or undesiring to engage in a behaviour [22,23].

Consistent with SDT, it has been repeatedly shown that autonomous motivation is more greatly associated with improved PA adherence than control-regulated motivation [20,21,24,25]. For instance, a study by Courtney et al. (2021) found that during the transition from adolescence to early adulthood, autonomous motivation was longitudinally associated with PA, suggesting its role in sustained, habitual PA [25]. In terms of CVD secondary prevention, this is additionally supported, showing more autonomously-motivated patients adhered better to PA recommendations during follow-up periods of CR [26,27]. However, motivation type may also be influenced by the setting and content of CR provided to patients, with prior findings reporting higher levels of controlled motivation in a conventional (HCP- monitored) CR setting, as compared to remotely-supported rehabilitation setting, where monitoring was shared between HCPs and patients [28]. As such, we are interested in assessing how motivation plays out in later stages of secondary prevention, i.e., when patients are primarily in a self-management stage. Specifically, we are interested in understanding the extent to which motivation is controlled versus autonomously-regulated in long-term PA engagement in secondary prevention.

Overall, we aim to assess patient preferences and determinants of PA engagement among a typical demographic (e.g., predominantly older age, male) Austrian cardiac sample in long-term secondary prevention. Additionally, we aim to gain insight into motivating factors for sustained PA by applying SDT to patients’ reasons for being physically active. Goal of these findings is to highlight determinants associated with sustained PA adherence, which may be particularly relevant for HCPs who aim to increase or maintain PA engagement in later stages of CVD secondary prevention. Overall, our findings provide useful insights into PA behaviour in secondary prevention.

## Methods

### Procedure

We conducted a cross-sectional survey using a postal questionnaire of closed- and open-ended questions. In our reporting of the study, we follow the Strengthening the Reporting of Observational Studies in Epidemiology (STROBE) guidelines [29]. Study recruitment took place from 01 May 2022 to 31 January 2023 at five cardiac rehabilitation (CR) centres located in four Austrian federal states (Carinthia, Lower and Upper Austria, Vorarlberg). One hundred questionnaires were administered to each of the five centres summing to a total of 500 distributed questionnaires. At the centres, internal staff identified patients who had completed an in- or outpatient phase II CR completion three years prior (starting from January 2019) from their internal patient documentation systems. Once these individuals were selected, staff sent out the questionnaires via the post to the patients’ home addresses retrieved from their patient documentation systems. In Austria, the phases of cardiac rehabilitation are defined as follows: phase I describes acute care; phase II consists of an intensive CR programme of 3–4 weeks duration in an inpatient setting or 4–6 weeks duration in an outpatient setting; phase III describes a 6–12 months outpatient CR programme with less frequent supervised sessions; and phase IV describes patients’ lifelong self-management of a recommended secondary prevention lifestyle [30]. After completion, respondents posted the questionnaires to our research institute using pre-paid envelopes. Participation in the study was voluntary, and as an incentive, patients could enter a random drawing to win one of four fitness trackers. In addition to the paper-and-pen survey, patients could opt to complete an online version (LimeSurvey © version 3.25.6) following a quick response (QR) code displayed on the front page of survey packet.

### Materials

An original pencil-and-paper questionnaire was designed and developed to address the specific aims of our study. Content of the questionnaire was based on qualitative interviews with 25 cardiac patients in 2020 (unpublished data) and underwent a rigorous, iterative testing process with members of the research team and seven cardiac patients. It additionally incorporated two standardised instruments: the EQ-5D-5L [31] and Rapid Assessment of Physical Activity [32]. The EQ-5D-5L measures self-assessed health status using both a descriptive system (mobility, self-care, usual activities, pain/discomfort and anxiety/depression) and a visual analogue scale (EQ-VAS). Dimension scorings consist of 1 (no problems) to 5 (extreme problems), and the EQ-VAS measures perceived health on a 0–100 scale (higher score representing better health). The RAPA consists of two parts, the RAPA-I, using a seven-question ordinal scale to assess active vs. suboptimal levels of aerobic PA and RAPA II which measures adherence to recommendations for muscle strengthening and flexibility exercises on a nominal scale.

The questionnaire was written in German and included four sections (demographic information, general health information, cardiac rehabilitation history and physical activity behaviour). In total it contained 32 items consisting of 17 multiple-choice, seven Likert scales, one visual analogue scale, six fill-in-the-blank questions and one open-ended response option. The content of the questionnaire can be accessed on the Open Science Framework platform (https://osf.io/hypmg).

### Inclusion criteria

Patients were included if they were ≥18 years old, had a diagnosed cardiovascular disease, had successfully completed a phase II (in- or outpatient) CR from one of the participating clinics in 2019 (approximately three years prior to the start of the study), were a resident in Austria and fluent in the German language. An upper age limit was not set as CVD primarily affects older-aged individuals [33] and we aimed to recruit as representative a sample of CVD patients as possible.

### Data storage

Questionnaires were retrieved up to two months after being sent. Data from the postal questionnaires was anonymised, entered onto LimeSurvey© platform, and subsequently exported as Microsoft Excel files and analysed in R (version 4.2.1). The paper questionnaires are archived in a secure location at the Ludwig Boltzmann Institute for Digital Health and Prevention, Salzburg, Austria, and the electronic forms are stored on an internal, secure computer network at the institute.

### Statistical analysis

Closed-ended (multiple choice, Likert, VAS scale and fill-in-the-blank) questions were analysed descriptively in terms of frequencies and percentages (n, %), central tendency (mean and median depending on item) and measure of spread (standard deviation [SD] or interquartile range [IQR]). Differences between men and women were assessed by Mann Whitney U test (two-tailed, p<0.05).

### Thematic analysis using self-determination theory

One open-ended question regarding patients’ motivations for exercising was analysed thematically using self-determination theory (SDT) as the guiding framework. SDT was chosen because it’s a well-supported theory in human motivation and extensive application in PA psychology, specifically, understanding individuals’ motivation for exercising [34]. In summary, SDT proposes that motivation lies on a spectrum ranging from *amotivation* (no motivation) to *intrinsic motivation* (out of enjoyment) with varying degrees of extrinsic motivation lying in between [23]. The analysis of our question follows the sub-theory of SDT called the organismic integration theory (OIT), which categorises and defines each component of motivation based on individuals’ locus of control vs. autonomy. Specifically, control-regulated motivation (i.e., regulated by others versus self) encompasses two extrinsic motivation categories, including the most controlled form of external motivation, external regulation (do something because others say so) to a less, but still controlled form, introjected regulation (acting out of guilt, pressure or contingencies). Autonomous motivation (i.e., self-regulated), on the other hand, encompasses two extrinsic motivation categories and intrinsic motivation. Here, the two autonomously-regulated extrinsic motivation categories are defined as identified regulation (behaving out of valued benefits of outcomes) and integrated regulation, the most autonomous form of extrinsic motivation (behaving because behaviour aligns with individuals’ sense of self or goals) [23]. According to SDT, autonomously regulated motivation is expected to relate to more long-term behaviour than controlled forms [22,23]. In categorising patients’ motivations according to the OIT, we followed a similar approach to Burn et al. (2019)–an inductive-deductive analysis of PA motivations through SDT using Braun and Clarke (2006) six steps to thematic analysis [35,36]. Specifically, the following steps were performed by the first author (HM); (step 1) an initial reading and transcribing of hand-written quotes from German to English onto an electronic platform (Microsoft Word, 2016). Translations of quotes were discussed with author SK to ensure meaning and significance of quotes were held. Transcripts were then read and re-read to allow for a familiarisation of data; (step 2) reading of transcripts line-by-line to generate initial codes deductively organised into SDT constructs. For consensus and clarity of coding, codes were iteratively discussed with author RC i.e., rationale for coding categorisation were discussed, any discrepancies addressed and a consensus drawn, and a re-coding of codes performed based on newly formulated definitions. If any discrepancies remained, this process was revisited; next (step 3), codes within the SDT constructs were inductively clustered together with those of similar codes to provide candidate themes; (step 4) an iterative process of reviewing and refining the themes to ensure themes worked in relation to the coded extracts and entire data set; (step 5) a final iterative re-defining and naming of each theme; (step 6) presentation of the themes in a coherent and logical, illustrative flowchart including number of occurrences of themes and overall number of occurrences of SDT constructs.

### Ethical considerations

The study was granted ethical approval from the following regional research ethics committees: Ethikkommission des Landes Kärnten (reference M2022-27), Ethikkommission der Medizinischen Fakultät der Johannes Kepler Universität (reference 1078/2022), and Ethikkommission für das Bundesland Niederösterreich (reference GS1-EK-4/795-2022). One regional research ethics committee (Ethikkommission des Landes Vorarlberg, reference EK-0.04–397) waived the need for formal ethical review. In conjunction with the postal questionnaire, patients received written information about the study. For this anonymous self-completed postal survey written consent was not required, but self-completion and return of the questionnaire were taken as implied consent and so approved by the research ethics committees.

## Results

### Demographics

In total, 117 patients responded to the survey (response rate: 23%). The sample consisted of mostly older (mean age 70±11 years), retired (76%), non- or ex-smoker (93%), male (76%) cardiac patients. For the majority of patients (82%), their cardiac history was attributed to coronary heart disease and/or heart failure (including angina pectoris, myocardial infarction, percutaneous coronary intervention, and coronary bypass surgery), and 13% had heart valve surgery.

Nearly half of patients (47%) had participated in an inpatient phase II CR, 43% in outpatient phase II CR and 3% in both an in- and outpatient phase II CR. Only 14% had additionally completed a phase III outpatient CR. Patients reported a median [IQR] perceived health score (EQ-VAS) of 80 [65,85] out of 100 *(higher scores indicating better health)*. In the EQ-5D categories mobility, self-care, usual activities, pain/discomfort and anxiety/depression the proportions of patients reporting problems (slight, moderate, severe or extreme problems combined) were 33%, 9%, 27%, 70% and 34%, respectively. Moreover, 47% of patients expressed confidence in their ability to manage their own health, 46% were rather confident, and 7% rather not confident. An overview of respondents’ demographic and clinical characteristics is displayed in Table 1.

**Table 1:** Respondents’ demographic and clinical characteristics.

Demographics	Subcategories	Output
Female, n (%) *(N = 116)*		28 (24)
Age (years), mean (SD) *(N = 113)*		70 (11)
Body mass index (kg/m^2^), mean (SD) (*N = 115)*		26.4 (3)
Employment status, n (%) *(N = 115)*	Employed	24 (21)
	Unemployed	1 (1)
	Retired	87 (76)
	Permanently unable to work	1 (1)
	Other	2 (2)
Family status, n (%) *(N = 114)*	Single	10 (9)
	Partnership	7 (6)
	Married/registered partnership	80 (70)
	Widowed	13 (11)
	Divorced/separated	4 (4)
People per household, median (IQR) *(N = 117)*		2 (2–2)
Smoking status, n (%) *(N = 115)*	Lifelong non-smoker	47 (41)
	Ex-smoker	60 (52)
	Since (years), mean (SD)	25 (16)
	Smoker	6 (5)
Country of birth, n (%) *(N = 117)*	Austria	113 (97)
	Other	4 (4)
Belonging to religious group, n (%) *(N = 117)*		68 (58)
Education, n (%) *(N = 117)*	Mandatory school (completion of nine years of school)	14 (12)
	High school degree (completion of 12 years of school)	14 (12)
	Apprenticeship with vocational school	38 (33)
	Technical or commercial school University or technical college degree	21 (18) 20 (17)
	Other	10 (9)
Household income (euros/month) after tax, n (%) *(N = 115)*	≤ 1200	5 (4)
	1,201–2,600	39 (34)
	2,601–4,000	25 (22)
	4,001–6,000	24 (21)
	6,001–8,000	4 (3)
	> 8,001	1 (1)
	No response	17 (15)
Medical cardiovascular history, n (%) *(N = 115)* *	Angina pectoris	10 (9)
	Coronary bypass surgery	14 (12)
	Cardiac arrhythmias	13 (11)
	Heart failure	20 (17)
	Heart valve surgery	15 (13)
	Myocardial infarction	36 (31)
	Percutaneous coronary intervention	61 (53)
	Pacemaker	4 (3)
	Other	15 (13)
Type of CR, n (%) *(N = 114)* *	Inpatient phase 2	53 (47)
	Outpatient phase 2	49 (43)
	Inpatient and outpatient phase 2	3 (3)
	Outpatient phase 3	16 (14)
	Long-term ambulatory rehabilitation	3 (3)
	Other rehabilitation provision	1 (1)
Perceived health status, EQ-VAS median (IQR) *(N = 116)*		80 (65–85)
Mobility, EQ-5D-5L, n (%) *(N = 117)*	No problems	78 (67)
	Slight problems	16 (14)
	Moderate problems	17 (15)
	Severe problems	5 (4)
	Unable/extreme	1 (1)
Self-care, EQ-5D-5L, n (%) *(N = 115)*	No problems	106 (92)
	Slight problems	6 (5)
	Moderate problems	1 (1)
	Severe problems	2 (2)
	Unable/extreme	-
Usual activities, EQ-5D-5L, n (%) *(N = 117)*	No problems	85 (73)
	Slight problems	22 (19)
	Moderate problems	7 (6)
	Severe problems	3 (3)
	Unable/extreme	-
Pain/discomfort, EQ-5D-5L, n (%) *(N = 117)*	No problems	35 (30)
	Slight problems	53 (45)
	Moderate problems	23 (20)
	Severe problems	5 (4)
	Unable/extreme	1 (1)
Anxiety/depression, EQ-5D-5L, n (%) (*N = 116)*	No problems	76 (66)
	Slight problems	28 (24)
	Moderate problems	7 (6)
	Severe problems	5 (4)
	Unable to function/extreme	-
RAPA I, n (%) *(N = 114)*	Sedentary	2 (2)
	Under-active	1 (1)
	Under-active regular–light activities	11 (10)
	Under-active regular	23 (20)
	Active^§^	77 (68)
RAPA II, n (%) *(N = 104)* *	Muscle strengthening guidelines	38 (37)
	Flexibility guidelines	72 (69)

* multiple responses permissible.

^§^ 30 minutes or more per day of moderate physical activities on 5 or more days per week; or 20 minutes or more per day of vigorous physical activities on 3 or more days per week.

CR, cardiac rehabilitation.

RAPA, Rapid Assessment of Physical Activity questionnaire.

### Physical activity behaviour

The sample was predominantly physically active in terms of aerobic training (68% as determined by the RAPA I). Flexibility goals as assessed by the RAPA II were achieved by 69% of patients and muscle strengthening goals by 37%. Nearly a quarter (24.7%) of patients achieved physical activity goals for *both* aerobic training and muscle strengthening. Overall, the median frequency and hours of PA per week were 4 [2,6] times a week and 4 [2,8] hours a week.

### Preferred type and place for physical activity

**Fig 1:**
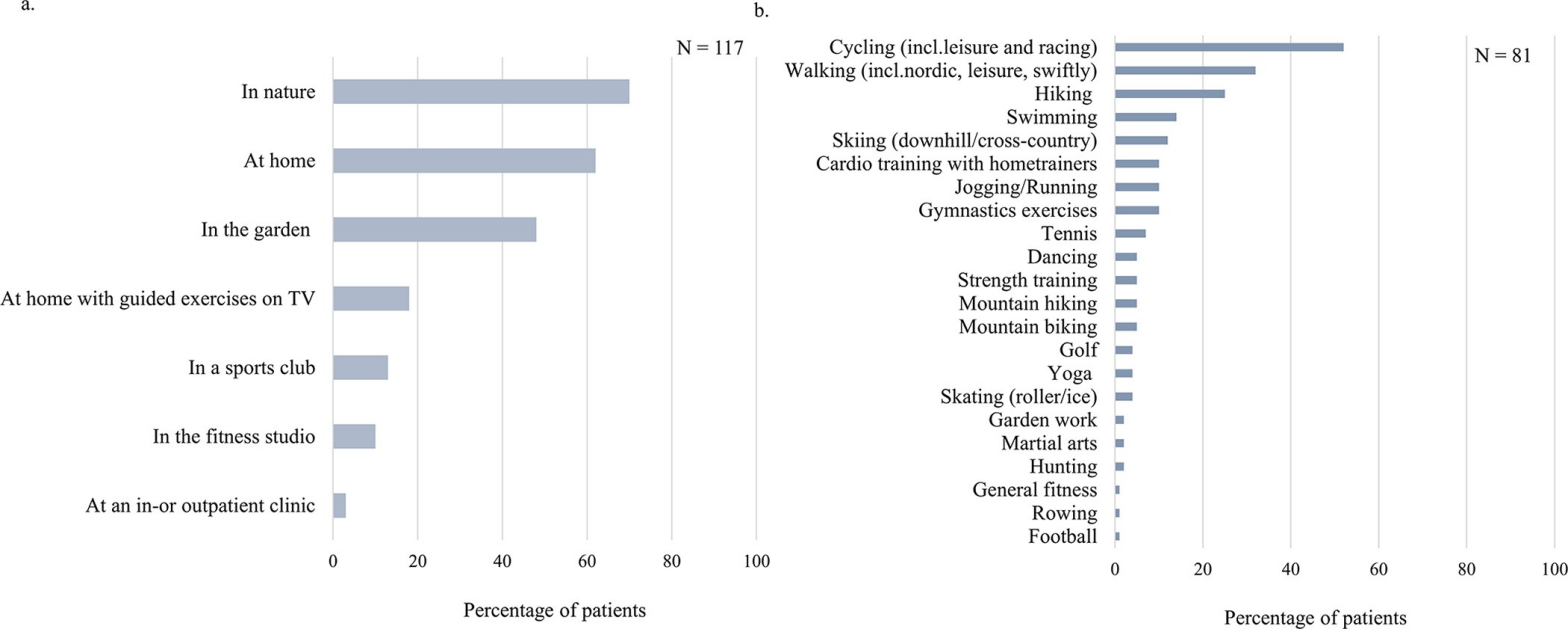
Physical activity location and type preferences. (A) Response rate to the questions ‘Where are you physically active?’. (B) Response rate to open-ended question ‘If you do play sports, what type(s) of sports do you engage in regularly? (Explanation: by sports we mean: ‘physical activity carried out according to certain rules, out of enjoyment of physical activity or games, for the purpose of physical activity’). *For A and B*, *multiple responses were permissible*.

We assessed patients’ preferred locations to engage in PA. The most popular PA location was outdoors in nature (70%), followed by at home (62%) and in the garden (48%). In comparison, less than a quarter (21%) reported being physically active in fitness studios *and/or* sports clubs (Fig 1A). Additionally, through an open-ended question, 81 (69%) patients expanded on the types of sport and physical activities they engaged in regularly. The most frequently reported sport was cycling, engaged in by over half (52%) of respondents. This was followed by walking (32%) and hiking (25%). On the other hand, traditional *team* sports were less frequently mentioned. Occurrences of tennis, football, martial arts, dancing and rowing were reported, but combined represented only ~15% of respondents. Furthermore, nearly all sports were cardio-based, whereas only 5% of patients reported engaging in muscle strengthening training (Fig 1B).

### Reasons and perceptions of physical activity

**Fig 2:**
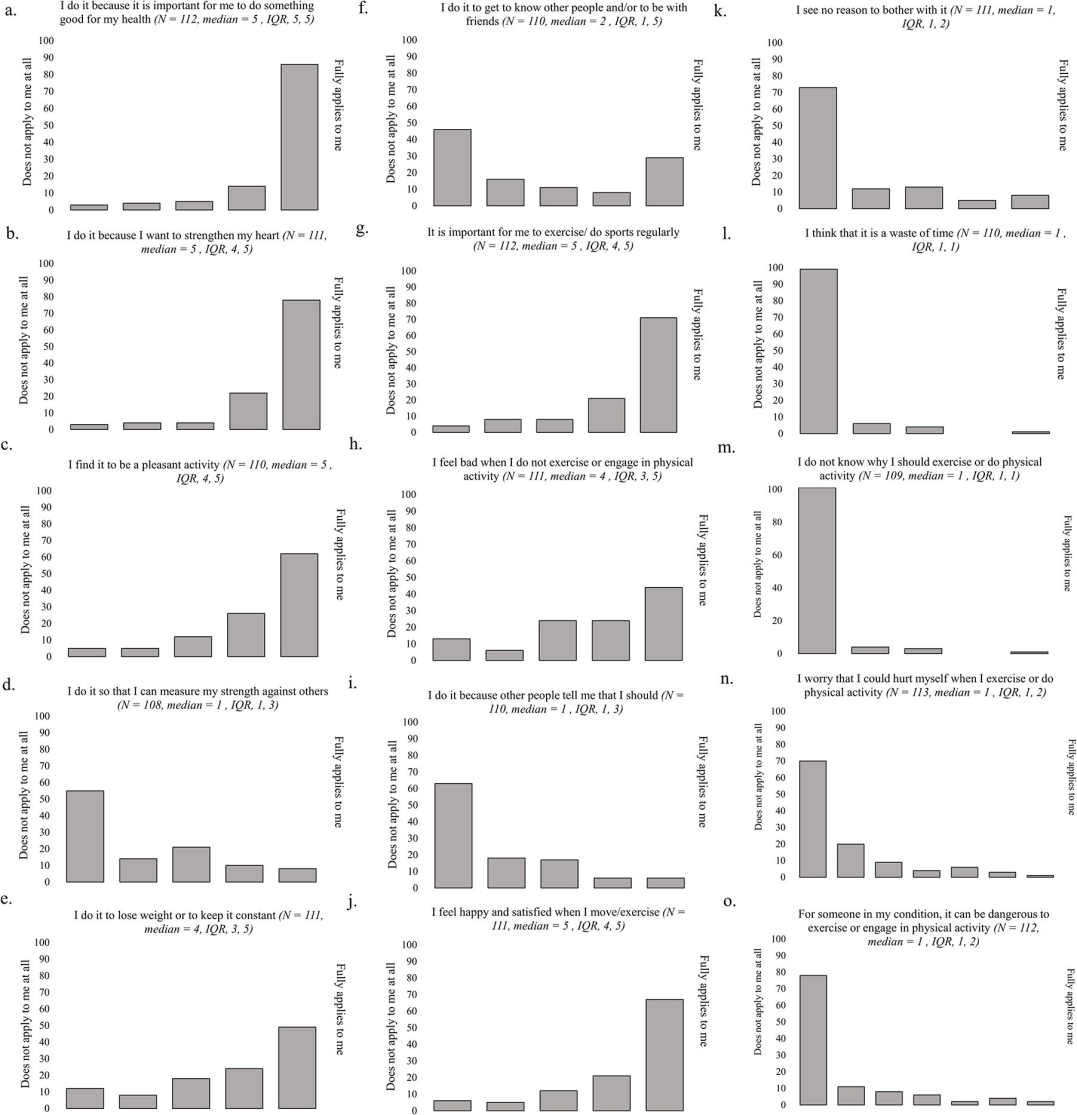
Response distribution of physical activity opinion statements. (A-M) Responses are provided on a 5-point Likert scale from 1 (does not apply to me at all) to 5 (applies fully to me) in response to the question ‘Please indicate to what extent the following statements apply to you’. (N-O) Responses are provided on a 7-point Likert scale from 1 (does not apply to me at all) to 7 (applies fully to me) in response to the question ‘To what extent do you agree with the following statements: Please think about physical activity and sports in relation to your heart conditions when you answer this question’. Frequency count (number of participants) is reported for all graphs.

Responses to patients’ perceptions and personal reasons for engaging in PA are displayed in Fig 2. Overall, the majority of patients (~74%) *strongly* expressed that their PA engagement stemmed from an importance to do something good for their health and a desire to strengthen the heart (Fig 2A and 2B). On the other hand, weight control was generally viewed positively, but less strongly as a reason for exercising, and competitiveness (skill-measuring against others) negatively i.e., was not generally perceived as a reason for PA (Fig 2E and 2D). In comparison, social factors (i.e., exercising to meet others) showed more varied results among patients, but for most patients did not serve as a reason for PA (Fig 2F).

Viewing PA as pleasant, satisfying and joyful was *strongly* expressed by ~58% of our respondents suggesting the majority of respondents exhibited intrinsic motivation for PA (Fig 2C and 2J). On the other hand, externally regulated factors—specifically because others say so—appears to play a marginal role in patients’ reasons for exercising (Fig 2I). Only 5% of patients *strongly* agreed that their reasons for exercising were impacted from other people saying so. In contrast, feeling bad for not exercising, which may reflect externally-regulated feelings of guilt or shame (e.g., introjected regulation) was reported to a larger extent (Fig 2H). Nearly 40% of patients *strongly* agreed that they felt bad when not being physically active. Thus, although most patients expressed intrinsic motivation for exercising, feeling bad for not exercising could reflect some extrinsically motivated drives.

Overall, patients expressed an understanding of the benefits and need for PA, as 90% of patients reported knowing why they should carry out PA and felt that it was not a waste of time (Fig 2L and 2M). However, when asked specifically if PA was important for the individuals themselves, only ~65% strongly agreed or felt that PA was something they should engage in (Fig 2G and 2K).

Lastly, most patients (~70%) did not report having a fear of exercising. This was expressed in terms of worry in hurting themselves and based on their medical condition (Fig 2N and 2O).

For all questions 2A to 2O, there were no statistically significant differences between men and women, except for question 2F (social factors), with a median of 2 versus 1, respectively (p = 0.03).

### Determinants of PA

**Fig 3:**
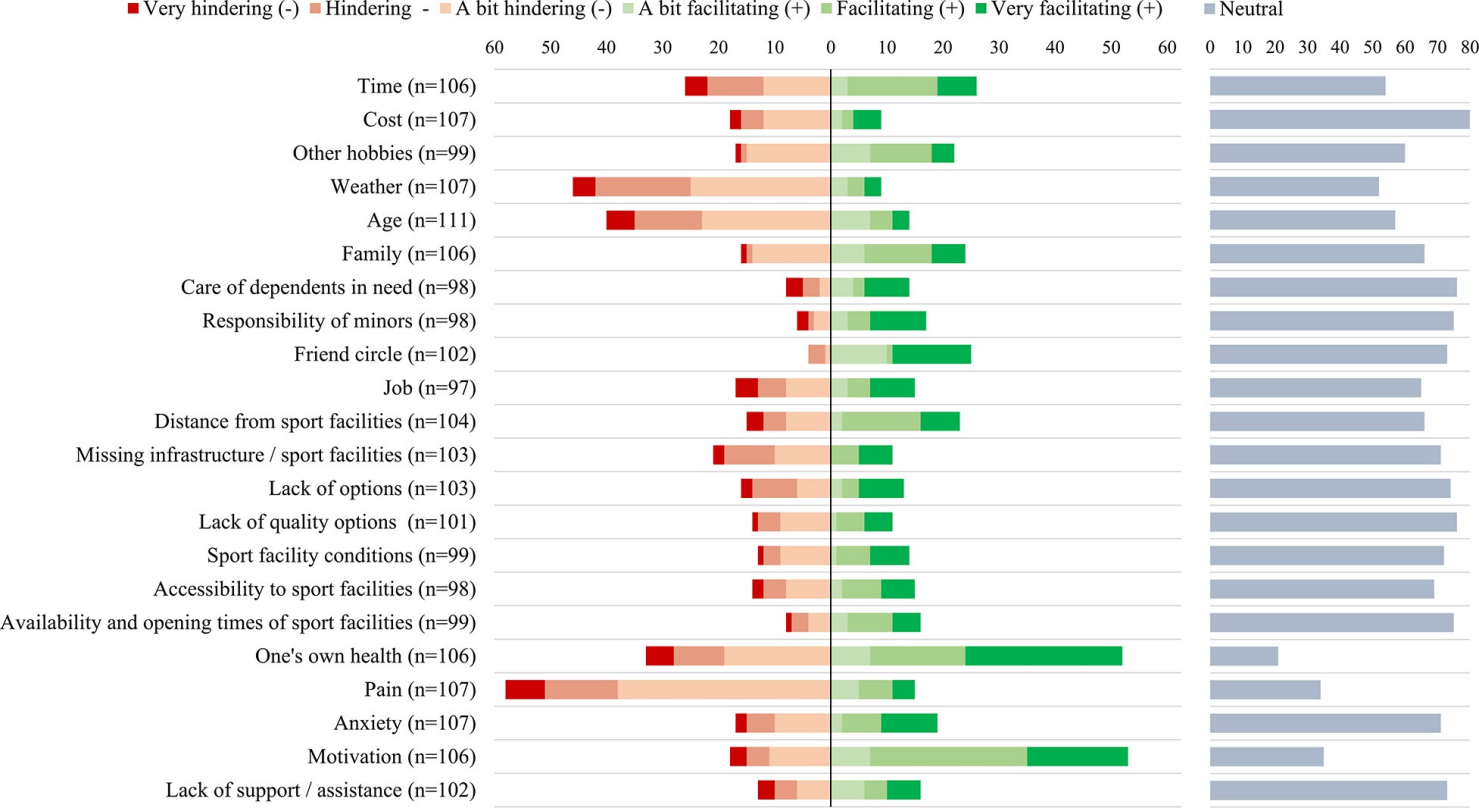
Directionality of the 22 queried determinants (potential facilitators and barriers) of physical activity behaviour. Responses are provided on a 7-point Likert scale ranging from ‘very hindering’ to ‘very facilitating’ in response to the question “To what extent do you find the following factors to personally be a hinderance or a facilitator for you in engaging in physically activity or doing sports?”. Shown are the direction and strength of each determinant (‘green’ = facilitating, ‘red’ = hindering, ‘grey’ = neutral) *(n = total sample size for question*, *y-axis = number of respondents)*.

Respondents rated the directionality and strength in which certain factors (e.g., sociodemographic, environmental, psychological) facilitated or hindered their engagement in PA (Fig 3). The top-3 influencing factors on PA were ‘one’s own health’, ‘pain’ and ‘motivation’, which served as determinants for 80%, 68% and 67% of patients, respectively. Furthermore, ‘weather’, ‘time’, and ‘age’ also served as determinants for PA, but to a lesser extent, by ~50% of patients.

In contrast, all other factors did not serve as determinants by most patients, with each factor only serving as a determinant for <40% of patients. Of these, ‘care of dependents’, ‘cost’, ‘responsibility of minors’, ‘lack of quality options’, and ‘availability and opening times of sport facilities’ were the least influencing factors on PA, as expressed by only roughly a quarter of patients.

### Motivating factors for engaging in PA

**Fig 4:**
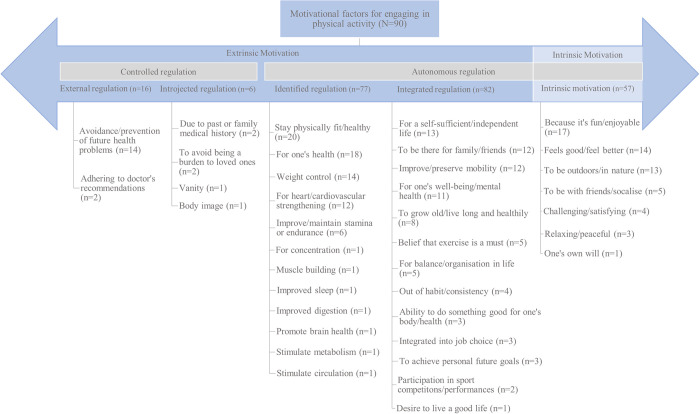
Patient-provided motivating factors for physical activity engagement categorised according to a framework of the self-determination theory of motivational continuum. Open-ended responses were provided to the question ‘State three things, circumstances or thoughts that personally most motivate you to engage in heart-healthy PA and keep you going’. *Multiple responses permissible per respondent (N = total sample size*, *n = number of response occurrences)*.

Ninety patients (77%) provided free-text responses expanding on the factors that personally motivate them to engage in heart-healthy PA. Overall, the top-3 most frequently mentioned reasons for PA engagement were ‘to stay physically fit/healthy’(n = 20), ‘for one’s own health’ (n = 18) and ‘because it’s fun/enjoyable’ (n = 17). The representation of these factors as organised according to the motivation continuum of the SDT are displayed in Fig 4. Results showed that majority of patients’ reasons for exercising (90%) fell under autonomous motivation, i.e., acting because of individual choice, values or own will. Only ~10% of motivating reasons were categorised under controlled regulation—motivation stemming for someone else’s control (e.g., exercising out of avoidance of adverse outcomes, guilt or perception/impact of others).

Under autonomous regulation, the majority of reasons that patients reported were associated with integrated regulation, i.e., motivating factors which related to individuals’ sense of self, values and goals. The most frequently occurring reasons for PA mentioned by patients were for sustaining a self-sufficient/independent life in old age, to be present for loved ones, for preserving mobility in age and for their well-being. Additional reasons relating to patients’ desires to increase length and quality of life, beliefs of PA, habits and personal goals were mentioned and categorised as integrated regulation.

Next, reasons for engaging in PA relating to the perceived benefits of PA were frequently expressed by patients and categorised under identified regulation. In terms of identified regulation, the most frequently perceived benefits of PA were related to fitness and health outcomes. Specifically, perceived benefits of physical health, general health, weight control and heart/cardiovascular strengthening were most frequently mentioned. Additional benefits of PA viewed by patients were relating to improved endurance, everyday functioning aspects (e.g., concentration, sleep, digestion, brain health) and muscle building.

Finally, intrinsic motivations for PA were present, but to a lesser occurrence than identified or integrated regulation reasons. The most frequently mentioned intrinsic motivations were those associated with viewing PA as a sense of enjoyment (e.g., fun and feeling good) and it relating to a desire and opportunity to be outdoors. Other factors that were mentioned were associated with ability to socialise, relax, as well as providing a sense of a satisfaction/challenge and one’s own will.

## Discussion

We conducted a cross-sectional multi-centre survey to assess cardiac patients’ behaviours, preferences and opinions of PA in long-term secondary prevention. Overall, we observed that our sample was generally physically active in terms of aerobic activities and displayed preferences for cardio, individual and outdoor sport activities (e.g., cycling, walking, hiking). Moreover, determinants for PA behaviour were mostly stipulated by physical factors (e.g., health and pain) and motivation—with autonomously regulated motivation playing a key role in PA engagement.

### Physical activity behaviour, preferences and beliefs

Overall, patients displayed a relatively high engagement in aerobic PAs in terms of minutes and frequency per week. However, only approximately a quarter (24.7%) of patients achieved the World Health Organisation (WHO)’s recommendations of combined aerobic and muscle strengthening training consistent with trends in the Austrian general population [37]. The need for increased muscle training is evident due to the positive combined outcomes of aerobic and muscle training observed. Specifically, the addition of resistance training on top of aerobic training leads to greater increments in physical fitness and muscle strength in CVD patients [38]. Moreover, muscle weakness is a strong predictor of premature death in CVD patients [39]. As such, our findings suggest that there may be a need for greater attention of muscle strengthening training in CVD secondary prevention. One potential practical implication is to initiate muscle strengthening interventions at the end of CR with the focus of supporting patients in transitioning to a home-based resistance routine. For example, a prior study, which utilised a social cognitive theory-based resistance training manual and Thera-Bands found increased self-efficacy, outcome expectations for and adherence to resistance training after CR, compared to standard exercise recommendation use [40]. Other possibilities could involve mobile health strategies, such as supportive push-messages and educational messages related to CVD management, which was found to be effective in increasing PA in a pilot randomised control trial [41]. Given that heart strengthening served as major motivational factor for PA engagement in our sample, interventions which educate patients on the beneficial effects of resistance training on heart strengthening, provide tailored training based on CVD condition [42], and exercise recommendations or materials that can be performed in a home setting may aide in increasing resistance training after a structured CR. For some patients, community-based strength training classes incorporating behavioural change strategies and peer support may be more fitting [43].

The majority of patients displayed PA location preferences for being outdoors, in their garden or at home, as well as activity preferences for individual, non-competitive sports. This reflects previous findings which report that retired adults spent more time in outdoor, natural environments, and older adults showed lower preferences for scheduled, competitive or team-based physical activities [44,45]. As our patient sample consisted primarily of older adults, our results could reflect age-related preferences for exercising. Practical implications might, thus, involve having clinicians or healthcare professionals recommend community programmes, such as walking groups or participation in non-competitive community-led events, such as fun-runs or group walks [46]. This could assist with goal-setting strategies and elicit positive effects associated with a sense of community feel. Additionally, given the high marital rates and older aged sample, couple-focused PA interventions could be a focus of secondary prevention, as the spouse often plays a significant social support role in later life [47,48]. This could allow for interventions that are adapted to the physical and social environment of the patients’ home. Furthermore, previous findings show that physically better and healthier patients prefer home- over centre-based cardiac rehabilitation [12], which could reflect the preferences we see for home-situated over sport facility-based PA among our sample which reported relatively high health-related quality of life. Thus, for this sample, promoting home-based exercises may encourage patients to exercise regularly, as it aligns with their preferred social-environmental setting and simultaneously limits barriers associated with gym-based exercising, such as travel time and membership costs [49]. However, for patients who are frail or less well, considerations may be required to ensure that at-home exercising is engaged in safely. Implications for this may include home-based PA coaches or novel digital approaches. For example, a computerized decision support system that could guide patients in their physical exercises may serve as a beneficial tool [50].

Overall, we found that patients displayed a strong understanding of why they should be physically active, indicating high awareness and knowledge of the positive outcomes associated with PA. However, fewer patients expressed strong agreement that they perceived being physically active was important for *themselves*. Thus, we highlight a potential dissociation for some between knowledge and internalised beliefs, and support prior studies that PA knowledge itself may not be sufficient for PA behaviour, and other approaches to increase a positive view of PA are needed [51]. Here, our suggestions include following guidelines for increasing PA, which is multi-faceted orientated. For example, Fletcher et al. (2018) addressed that promoting PA should be targeted at the individual, educational/community and societal level. This included actions such as personalised exercise recommendation, self-monitoring and reducing barriers to PA (at the individual level), PA promotion during outpatient visits by physicians and community-led initiatives (at the educational/community level) and better PA-promoting infrastructure and green spaces (at the societal level) [52].

### Determinants of physical activity engagement

We found that patients perceived their own health, pain and motivation as main determinants for PA engagement. For the majority of patients, their health–whether good or bad (self-reported)–was perceived to facilitate their engagement in PA. Thus, patients in our sample may have generally viewed their health positively in terms of supporting an active lifestyle (i.e., providing them with the capacity and ability to engage in physical activities). This would align with the general high perception of health-related quality of life status we report in our results, and highlight prior findings that greater perception of health is correlated with more PA engagement [53]. Similarly, health may function as facilitator for PA primarily out of motivation for improved or better health, as health benefits of PA are indicated to play a significant role in cardiac patients’ engagement in PA [16,17]. On the other hand, poor health may hinder PA engagement if health constraints limit ability and/or participation in PA [54,55]. In this case, PA prescription may be particularly useful in bridging communication between health care providers and patients, and ensuring PA is appropriate, safe and fitting to patient needs [56]. Here, we suggest the use of shared decision making between an HCP and patient to ensure that PA exercises align to patients’ identified health goals and values [57,58]. In our study, we found that along with physical and general health outcomes, patients expressed specific goals of weight control, heart strengthening, and quality of life among others. Thus, practical implications may involve educating patients and recommending physical activity regimes that target these goals.

Pain similarly served as an important determinant of PA engagement, but functioned primarily as a barrier to PA engagement, as is mirrored in recent literature [54,59]. Specifically, an association between musculoskeletal pain and greater mobility disability highlights the role that pain plays in reduced bodily movement and performance of daily activities [60]. Despite this, a few patients rated pain as facilitating for PA engagement, which may indicate motivation to engage in PA for reasons of pain reduction or improved physical function goals [61]. Interestingly- and somewhat surprisingly, in our study, fear-related factors (e.g., worry of injury, perceived danger, anxiety) were not greatly present, despite documentation in similar research reporting a close link between pain and fear [62]. In particular, prior studies highlight fear of PA poses a major challenge in PA engagement among cardiac patients [63,64]. Potential reasons for the low-reported mental distress in our sample may be a result of having attended CR, where they learned about the needs, benefits and safe manners to be physically active. This would support prior findings indicating an association between CR attendance and reduced odds of kinesiophobia (i.e., fear of movement) [64]. Thus, our data call for further intensifying the current practice of implementing PA interventions which simultaneously target physical and mental factors to enhance PA adherence in secondary prevention.

Finally, motivation additionally served as an important determinant for PA, and was mostly viewed as facilitating for PA engagement. Given the general high engagement in aerobic training in our sample, our findings echo those of earlier ones that motivation is important for engagement in PA [18,65]. On the other hand, some patients marked motivation as hindering for PA engagement, which likely suggests a lack or inability to motivate themselves to be active. This may be attributed to a variety of factors including loss of interest, inability to see benefits or health constraints [66]. In this case, goal-setting strategies may be particularly effective for increasing PA behaviour, as has been supported in a prior systematic review [67]. Practical implications could thus involve self-tracking methods to monitor progress or supported guidance from a healthcare provider or sports coach. Given that goal-setting interventions were observed to be effective regardless of the delivery mode [67], this sheds light on the possibility of digital tools to serve as a practical medium for goal tracking.

Although age, time and weather were reported to a lesser extent as determinants for PA, they still played a role in PA engagement for roughly half of patients. Thus, these factors still warrant observation and may contribute to PA adherence for certain demographic groups. For instance, time and age factors suggest that PA interventions should be adapted for a variety of age- or scheduling needs (e.g., older age, retired vs. working adults, etc) [44,68]. Moreover, as weather factors may influence PA engagement, efforts to encourage PA in a variety of weather and climate conditions may help to increase adherence among patients, and may be especially needed during colder or rainy seasons [69]. Promoting indoor physical activities or adapting PA intensity to seasonal changes may encourage PA in seasons with low PA, such as winter [70].

Overall, the least influencing factors on PA were caring responsibilities (e.g., dependents or minors) and resource-related factors (e.g., cost, quality or availability of sport facilities). Given our general older age, retired sample, this may reflect generational-specific influences. Specifically, in that responsibility-related barriers (e.g., family, job) may play less of role in PA engagement for older adults, allowing for more flexibility in terms of time commitment and scheduling [68]. Moreover, the largely neutral stance of resource-related factors suggests that patients mostly do not perceive sport facilities (e.g., options, quality, availability, etc) as influencing for PA and echo the aforementioned preferences we see for outdoor or home-based PA locations over sport centres. This may lend itself well for PA interventions, such as digital health interventions, which can be engaged in remotely, self-sufficiently, and in the comfort of patients’ home or outdoor environment [71]. Interestingly, we did not find that social support factors (e.g., family and friends) contributed greatly to PA engagement, which again may indicate higher preferences for individualistic rather than team-based activities in this demographic group [44]. Overall, our findings highlight the importance of considering generational and lifestyle needs and preferences in the design of PA interventions.

### Motivation for PA using self-determination theory

In terms of patients’ motivating factors for PA, our results display an overwhelming amount originating from autonomously regulated motivation. As our respondents had been in CR approximately three years prior to our survey and were therefore in a phase of lifelong lifestyle change, i.e., long-term secondary prevention, our results support other findings suggesting that intrinsic motivation and more autonomously regulated extrinsic motivation contribute to long-term behaviour maintenance [20,72].

The most frequently mentioned motivating factors, namely, enjoyment, positive health outcomes, and alignment with goals and sense of self (e.g., remaining self-sufficient, present for family, for one’s well-being, aging healthily) are consistent with previous findings of PA adherence among cardiac patients. Specifically, Fraser et al. (2022) reported that cardiac patients were motivated to be physically active out of reasons for regained independence, improved mental and physical well-being, as well as prolonged life [16]. Additionally, Warehime et al. (2020) reported that improvements in individual health and changes to well-being motivated heart failure patients to engage in long-term PA adherence [65]. Interestingly, we also reported a frequent mention of being in nature and outdoors as a motivating reason for PA in our sample. This is aligned with our aforementioned closed-ended question results pertaining to patient preferences for exercising outdoors and further strengthens the role of nature as a motivating factor in PA engagement in our sample.

In contrast, we do not report many controlled extrinsic motivations (e.g., others saying so, out of reasons to escape guilt) in our findings. However, out of the controlled motivations we see, the most frequently mentioned factor was exercising out of an avoidance or prevention of adverse health problems. Although avoidance or prevention may be facilitating for PA, reasons stemming from a fear of disease progression may be linked to distress and negative emotional responses in chronic disease patients [73]. Thus, it is important that individuals who are mainly motivated to engage in PA primarily out of avoidance reasons are also provided with necessary emotional support to cope with illness distress if needed.

Overall, the general few frequencies of controlled motivation we see may be related to the stage of secondary prevention in which patients found themselves. It is likely that patients were initially more extrinsically motivated to be physically activate due to physician or CR recommendations, but over time these motivations became more internalised. This would be supportive of prior findings, which show increased autonomy satisfaction during CR predicted increased intrinsic motivation and in turn long-term PA [74]. On the other hand, this may suggest that patients who struggle to internalise behaviour regulations during initial stages of secondary prevention, may have difficulty adhering to PA in the long-term. As such, specific attention may be needed to support those who struggle to internalise behaviour regulations. Practical implications may include assessing patients’ sense of autonomy, competence and relatedness, as well as self-determined motivation during CR, as it can provide potential indication of long-term PA adherence [74,75].

Despite the observations we see in our study, cardiac patients do not generally maintain adherence to PA recommendations following CR [7,10,11,76]. Possible reasons for this are that patients are not provided with the resources, knowledge or time to integrate these behaviours into their daily lives. To combat this, habit-formation techniques (e.g., guidance, cues, reminders, discussed shared experiences and action planning) may be of particularly use in increasing adherence [77]. Thus, improvements in long-term PA interventions could be focused on increasing patient autonomy and capacity to establish PA habits in conjunction with service models that cater for patients long-term, such as provision of ongoing supervised exercise therapy and digital/telerehabilitation offers.

### Limitations and future directions

Our study was mainly limited by the self-selected nature of the sample, leading to possible response bias towards more physically active and healthy cardiac patients. Additionally, as the study was self-administered and anonymous, we were unable to follow-up on patient responses, which would have been particularly beneficial for understanding influences on response reasoning behind determinant selection and motivating factors. Moreover, this meant that we could not verify that the posted individuals filled out the questionnaire themselves or if anybody influenced their response decision (e.g., caregivers, family members). Use of the CR clinics to send the questionnaires did help ensure that the questionnaires were correctly addressed to patients’ addresses, however, the absence of a present researcher meant that a direct observation of questionnaire completion or the ability to intervene if participants had questions/difficulties was not possible. We attempted to counter this by using two main strategies. Firstly, the questionnaire underwent a prior rigorous, iterative testing process with seven cardiac patients to pilot test understanding of the questionnaire content in a small sample. Secondly, we provided contact information (email and phone number) of the research team, in case any patients wished to contact us for assistance or information. We acknowledge a limitation in that, despite having an older age sample, we did not assess participants’ cognitive level nor could accurately assess cognitive level given the study design. Given the results of the EQ-VAS, it suggests our sample mainly consisted of patients with relatively good health-related quality of life, but future work would benefit from assessing cognitive level in such an older-aged patient sample.

Despite our limitations, we gained important insights into a rather unexplored area of PA motivation in long-term CVD secondary prevention, and through the use of a postal questionnaire, were able to also reach those less technologically affined, typically older adults. Future directions could focus on conducting in-depth qualitative interviews and focusing on recruiting harder-to-reach patients (e.g., physically inactive and/or less healthy). Furthermore, our findings highlight the importance of increasing autonomous motivation for long-term PA engagement in secondary prevention. Future work could be focused on implementing specific SDT-theory techniques in long-term secondary prevention. For instance, Teixeria et al. (2020) employed an iterative expert consensus procedure to develop a classification list of 21 SDT-driven motivation and behaviour change techniques for use in health contexts [75]. The classification is based on the three underlying psychological constructs of SDT, namely increasing autonomy, competence, and relatedness, which in turn, are intended to promote autonomous motivation [75]. These include techniques, such as promoting self-monitoring, electing meaningful (e.g., personal, tailored) rationale for behaviour change, creating a concrete plan of action and eliciting perspectives on the condition or behaviour [75]. As such, the integration of health behaviour change techniques that are in alignment with existing patient-desired health outcomes (e.g., longevity, independence, mobility) may encourage greater autonomous motivation and in turn adherence to PA.

## Conclusions

The success of PA-promoting interventions in the secondary prevention of CVD is based on the needs and preferences of cardiac patients. Our results indicate that individual, cardio and outdoor activities are especially well received by cardiac patients and could be of particular use in designing PA interventions. Additionally, although increasing general PA levels should be of primary importance, our findings call on a need for promoting muscle strength training in secondary prevention. Moreover, our findings highlight that physical factors (e.g., health, pain) and motivation–especially autonomous motivation–play important roles in engagement of PA in long-term secondary prevention. Thus, PA interventions which foster autonomous motivation–specifically through alignment with patient preferences and/or health or pain-related goals–may lead to increased adherence to PA, even in later stages of the CR pathway.
